# Tetanus insensitive VAMP2 differentially restores synaptic and dense core vesicle fusion in tetanus neurotoxin treated neurons

**DOI:** 10.1038/s41598-020-67988-2

**Published:** 2020-07-02

**Authors:** Rein I. Hoogstraaten, Linda van Keimpema, Ruud F. Toonen, Matthijs Verhage

**Affiliations:** 1 Department of Functional Genomics, Center for Neurogenomics and Cognitive Research, Vrije Universiteit (VU) Amsterdam and University Medical Center Amsterdam, de Boelelaan 1087, 1018 HV Amsterdam, The Netherlands; 2 Clinical Genetics, Center for Neurogenomics and Cognitive Research, Vrije Universiteit (VU) Amsterdam and University Medical Center Amsterdam, de Boelelaan 1087, 1018 HV Amsterdam, The Netherlands; 3 Sylics (Synaptologics BV), PO Box 71033, 1008 BA Amsterdam, The Netherlands

**Keywords:** Neuroscience, Cellular neuroscience, Molecular neuroscience, Synaptic transmission

## Abstract

The SNARE proteins involved in the secretion of neuromodulators from dense core vesicles (DCVs) in mammalian neurons are still poorly characterized. Here we use tetanus neurotoxin (TeNT) light chain, which cleaves VAMP1, 2 and 3, to study DCV fusion in hippocampal neurons and compare the effects on DCV fusion to those on synaptic vesicle (SV) fusion. Both DCV and SV fusion were abolished upon TeNT expression. Expression of tetanus insensitive (TI)-VAMP2 restored SV fusion in the presence of TeNT, but not DCV fusion. Expression of TI-VAMP1 or TI-VAMP3 also failed to restore DCV fusion. Co-transport assays revealed that both TI-VAMP1 and TI-VAMP2 are targeted to DCVs and travel together with DCVs in neurons. Furthermore, expression of the TeNT-cleaved VAMP2 fragment or a protease defective TeNT in wild type neurons did not affect DCV fusion and therefore cannot explain the lack of rescue of DCV fusion by TI-VAMP2. Finally, to test if two different VAMPs might both be required in the DCV secretory pathway, *Vamp1* null mutants were tested. However, VAMP1 deficiency did not reduce DCV fusion. In conclusion, TeNT treatment combined with TI-VAMP2 expression differentially affects the two main regulated secretory pathways: while SV fusion is normal, DCV fusion is absent.

## Introduction

Soluble *N*-ethylmaleimide-sensitive factor attachment receptor (SNARE) complex formation is essential for secretion in the two main regulated secretory pathways in neurons, synaptic vesicle (SV) and dense core vesicle (DCV) exocytosis^1,2^. While the SNARE complex that typically drives SV fusion, consisting of syntaxin-1, synaptosomal associated proteins of 25 kDA (SNAP-25) and vesicles associated membrane protein 2 (VAMP2 or synaptobrevin 2), has been studied in great detail^2,3^, the cognate SNARE proteins for DCV fusion are still poorly characterized.

The seven genes of the VAMP family all encode proteins that contain a SNARE and transmembrane domain^2,4,5^. All VAMP proteins, except VAMP5, are reported to form functional SNARE complexes^5^. VAMP2 is the most abundant and widely distributed VAMP protein in the brain^6,7^. In hippocampal neurons, deletion of VAMP2 expression (VAMP2 knock out (KO)) impairs calcium-dependent SV fusion in most but not all neurons^8,9^. Inhibiting VAMP2 expression in cortical neurons using short-hairpin RNA reduces DCV exocytosis^10^. VAMP1 (synaptobrevin 1) is highly expressed in the spinal cord and less abundant in the brain compared to VAMP2^11^, but may be the main VAMP isoform in certain brain areas^7,12^ or neuronal subtypes^9,13^. In a subset of VAMP2 KO neurons which highly express VAMP1, SV fusion is less impaired^9^. Loss of VAMP1 reduces synaptic transmission at the neuromuscular junction^11^. Selective knockdown of VAMP1 in rat trigeminal neurons reduces release of calcitonin gene-related peptide (CGRP), a dense core vesicle cargo peptide, whereas selective cleavage of VAMP2 with botulinum neurotoxin B (BoNT/B) does not, suggesting VAMP1 to be the main isoform in these neurons^14,15^. Secretory granule exocytosis is only mildly affected in VAMP2 KO chromaffin cells and gene-inactivation of VAMP3 (cellubrevin) has no effect. However, granule exocytosis is severely impaired in the absence of both VAMP2 and VAMP3, suggesting functional redundancy^16^. VAMP3 expression is undetectable in neurons^8,17^ but highly expressed in glial cells where it mediates secretion of NPY containing vesicles^18^. Taken together, although VAMP2 is the major isoform for SV and DCV exocytosis, VAMP3 can partly take over its function in chromaffin cells and VAMP1 drives at least some SV fusion in neurons as well as CGRP release from trigeminal ganglionic neurons. It is unknown if all DCV fusion in the brain is mediated by VAMP2 or if functional redundancy between isoforms occurs.

Here, we studied the role of VAMP proteins in neuronal DCV exocytosis in single hippocampal neurons. Neurons were treated with tetanus neurotoxin (TeNT), which specifically cleaves VAMP 1, 2 and 3 but not the other four VAMP proteins^10,19–23^. TeNT disrupted both SV and DCV fusion. Expression of a tetanus-insensitive (TI) version of VAMP2 (VAMP2 Q76V, F77W^24^) restored SV fusion. However, expression of TI-VAMP1, TI-VAMP2 or TI-VAMP3 did not restore DCV exocytosis while both TI-VAMP1 and TI-VAMP2 travel with DCVs through neurites. To study the potential role of VAMP1 in DCV exocytosis we used *vamp1lew (*lethal wasting, hereafter referred to as *vamp1*^*–/–*^) mouse that lacks VAMP1^25^. DCV fusion in *vamp1*^*–/–*^ neurons was unaffected. Hence, TeNT treatment combined with TI-VAMP2 expression differentially affects SV and DCV fusion and may be used as a tool to selectively inhibit DCV fusion, leaving SV fusion unchanged.

## Results

### TeNT efficiently cleaves VAMP 1, 2 and 3, and abolishes DCV exocytosis

**Figure 1:**
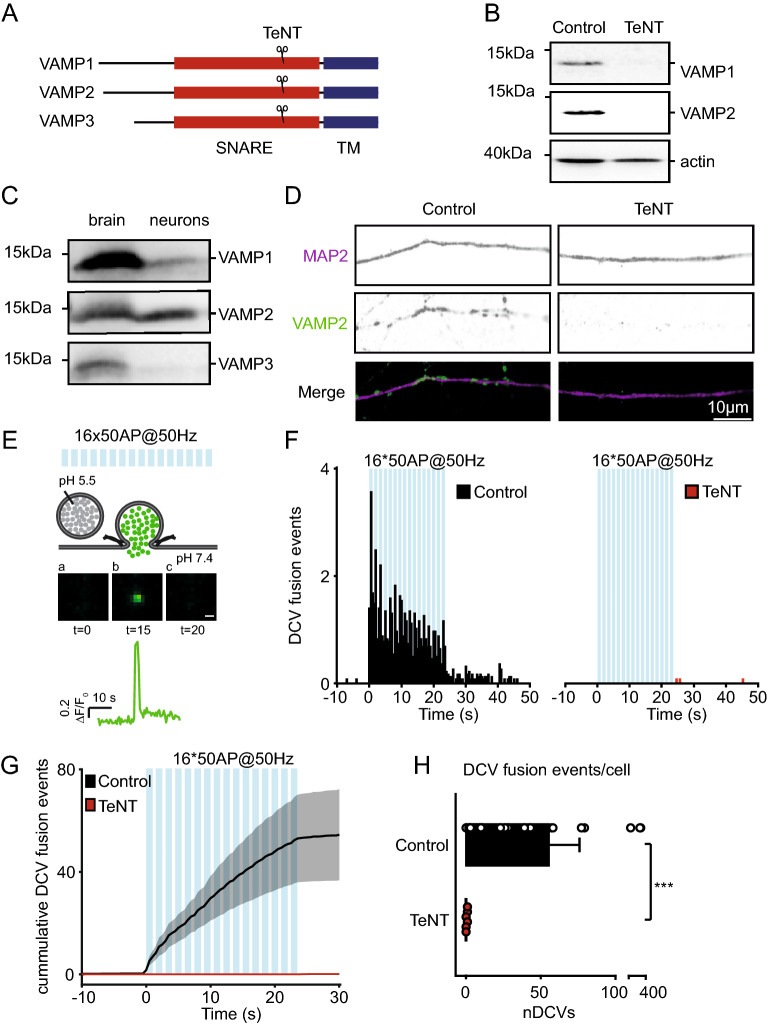
TeNT efficiently cleaves VAMP1, 2 and 3, and abolishes DCV exocytosis in hippocampal neurons. (**A**) Schematic representation of VAMP1, 2 and 3 proteins with TeNT cleavage site. Indicated are the transmembrane (TM, blue) and SNARE (red) domains. (**B**) Western blot of cortical neurons (DIV 16), infected with a control construct or TeNT at DIV 14, incubated with antibodies against VAMP1 or VAMP2. Actin was used as loading control (full length gels are shown in Figure S2). (**C**) Western blot of whole brain and cortical neurons (DIV 13), incubated with antibodies against VAMP1, VAMP2 or VAMP3 (original blots are shown in Figure S3). (**D**) Representative images of a neurite stretch at DIV 14 of control construct and TeNT infected (DIV 10) hippocampal neuron, stained for dendritic marker MAP2 (magenta) and VAMP2 (green). (**E**) Schematic representation of the method to detect DCV exocytosis in neurons infected with NPY-pHluorin. Electrical stimulation (16 trains of 50 AP at 50 Hz (blue bars) interspaced by 0.5 s) elicits DCV fusion with the plasma membrane, de-quenching NPY-pHluorin through an increase from pH 5.5 (in the DCV lumen) to pH 7.4. Before stimulation, NPY-pHluorin is quenched (a). During stimulation DCVs fuse with the plasma membrane visualized by a rapid increase in fluorescence (b) followed by a rapid decrease through cargo release or fusion pore closure and re-acidification (c). Scale bar 1 µm. Trace indicates F/F0 of this event. (**F**) Histogram of DCV fusion events in control (black) and TeNT infected (at DIV 9–10, red) hippocampal neurons imaged at DIV 14 (blue bars indicate 16 trains of 50 APs at 50 Hz interspaced by 0.5 s). (**G**) Cumulative plot of DCV fusion events in control construct and TeNT infected neurons. Shaded area represents SEM. (**H**) Average DCV fusion events per cell in control (n = 21, N = 3) and TeNT (n = 21, N = 3) infected neurons. Mann–Whitney *U* test: ***p = 4.5* 10^−7^. Bars represent mean + SEM. Detailed statistics are shown in Supplementary Table S1.

TeNT is known to specifically cleave VAMP 1, 2 and 3 (Fig. 1A), but not VAMP 4, 5, 7 and 8^10,19–23^. We confirmed that lentiviral infection of TeNT light chain in cortical neurons efficiently cleaves VAMP1 and VAMP2 (Fig. 1B), the only two TeNT-sensitive VAMP proteins expressed in these neurons (Fig. 1C), as shown before^8,17^. Hippocampal neurons, stained for VAMP2 and dendritic marker MAP2, showed no VAMP2 staining after lentiviral infection with TeNT (Fig. 1D). These results confirm efficient cleavage of VAMP proteins by TeNT lentiviral expression.

To study DCV exocytosis, we expressed a well validated DCV fusion reporter (NPY-pHluorin^26–32^) in hippocampal neurons. Trains of action potentials, known to produce maximal DCV fusion (16 times 50 action potentials (AP) trains at 50 Hz interspaced by 0.5 s), elicited DCV exocytosis detected as abrupt appearance of fluorescent NPY-pHluorin puncta (Fig. 1E)^26–32^. Co-infection with NPY-pHluorin and TeNT (4–5 days before imaging, identified by IRES-mCherry expression tag) disrupted DCV exocytosis (DCV fusion events control: 57 ± 19, TeNT: 0.14 ± 0.08; Fig. 1F–H) but did not alter the number or sub-cellular distribution of DCVs (Supplementary Fig. S1). In conclusion, TeNT cleaves VAMP 1, 2 and 3 and disrupts DCV exocytosis in hippocampal neurons.

### TeNT insensitive (TI)-VAMP2 efficiently restores SV exocytosis upon TeNT treatment

**Figure 2:**
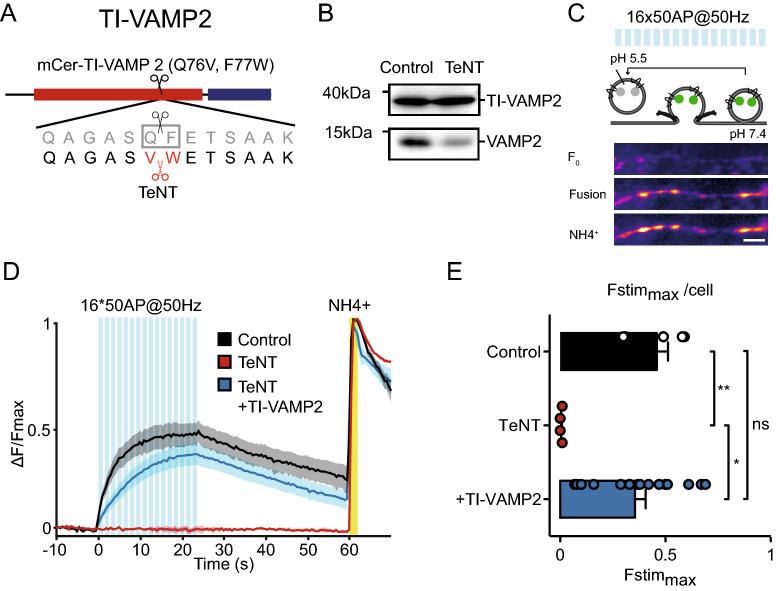
TI-VAMP2 rescues SV fusion upon TeNT treatment. (**A**) Schematic representation of TI-VAMP2. Q76V, F77W substitutions interfere with TeNT cleavage. N-terminal mCerulean (mCer) allows detection of the construct during live-cell experiments. (**B**) Western blot of neuronal lysate (DIV 16) infected with TI-VAMP2 (DIV 2) and a control construct or TeNT (DIV 14), incubated with VAMP2 antibody (original blots are shown in Figure S4). (**C**) Schematic representation of the method to measure synaptic vesicle exocytosis in neurons infected with synaptophysin-pHluorin (sypHy). Electrical stimulation (16 trains of 50 AP at 50 Hz (blue bars) interspaced by 0.5 s) elicits SV fusion with the plasma membrane, de-quenching sypHy through the increase in pH from 5.5 (in the SV lumen) to 7.4. Before stimulation, sypHy is quenched (F_0_). During stimulation, SVs fuse with the plasma membrane which is visualized by gradual increase in fluorescence (Fusion). Upon NH_4_^+^ perfusion, the total sypHy labeled SV pool is visualized (NH_4_^+^). Scale bar 5 µm. (**D**) ΔF/Fmax sypHy (infected at DIV 3–4) signal before, during and after electrical stimulation (blue bars) in control, TeNT (infected at DIV 9), and TeNT (infected at DIV 9) + TI-VAMP2 (infected at DIV 2 and 9) neurons imaged at DIV 11. Yellow bar represents NH4^+^ superfusion, which de-quenches all labeled SVs and reveals the total pool (used as max in the ΔF/Fmax plot). Shaded area represents SEM. (**E**) Average Fstim_max_ (peak in the ΔF/Fmax graph, see D) for control (n = 6, N = 2), TeNT (n = 4, N = 2) and TeNT + TI-VAMP2 (n = 17, N = 2) neurons. Kruskal–Wallis with Dunn's correction: *p = 0.0116, **p = 0.0067, non-significant (ns) *p* > 0.99. Bars represent mean + SEM. Detailed statistics are shown in Supplementary Table S1.

To determine whether VAMP2 is sufficient for regulated exocytosis, we introduced TeNT insensitive (TI)-VAMP2 in TeNT treated neurons. TeNT cleaves the bond between glutamine 76 and phenylalanine 77 in VAMP2^23^. Mutation of these sites to valine and tryptophan, respectively, renders VAMP2 resistant to TeNT cleavage (Fig. 2A)^24,33,34^. TI-VAMP2 (VAMP2 Q76V, F77W) was N-terminal tagged with mCerulean^24^ which enabled detection of infected neurons during live cell experiments.

We first confirmed the functionality of TI-VAMP2 in SV exocytosis using synaptophysin-pHluorin^35^. Neurons were infected with synaptophysin-pHluorin and with TeNT, TeNT and TI-VAMP2, or with a control construct. Lentiviral infection of TeNT efficiently cleaved endogenous VAMP2 but not TI-VAMP2, which was expressed at a similar level as endogenous VAMP2 (Fig. 2B). Upon the same trains of action potentials as used before for DCV exocytosis (16 trains of 50 AP at 50 Hz), SV exocytosis was detected as an increase in fluorescence at puncta (Fig. 2C). TeNT treatment abolished SV fusion (Fig. 2D, red line) as shown before^23,36,37^. Co-infection of TI-VAMP2 restored SV exocytosis (Fstim_max_ control: 0.46 ± 0.05, TeNT: 0.01 ± 0.0, TeNT + TI-VAMP2: 0.36 ± 0.05; Fig. 2E). This indicates that TI-VAMP2 is functional and sufficient to support SV exocytosis.

### Neither TI-VAMP1, TI-VAMP2 nor TI-VAMP3 supports DCV exocytosis in TeNT treated neurons

**Figure 3:**
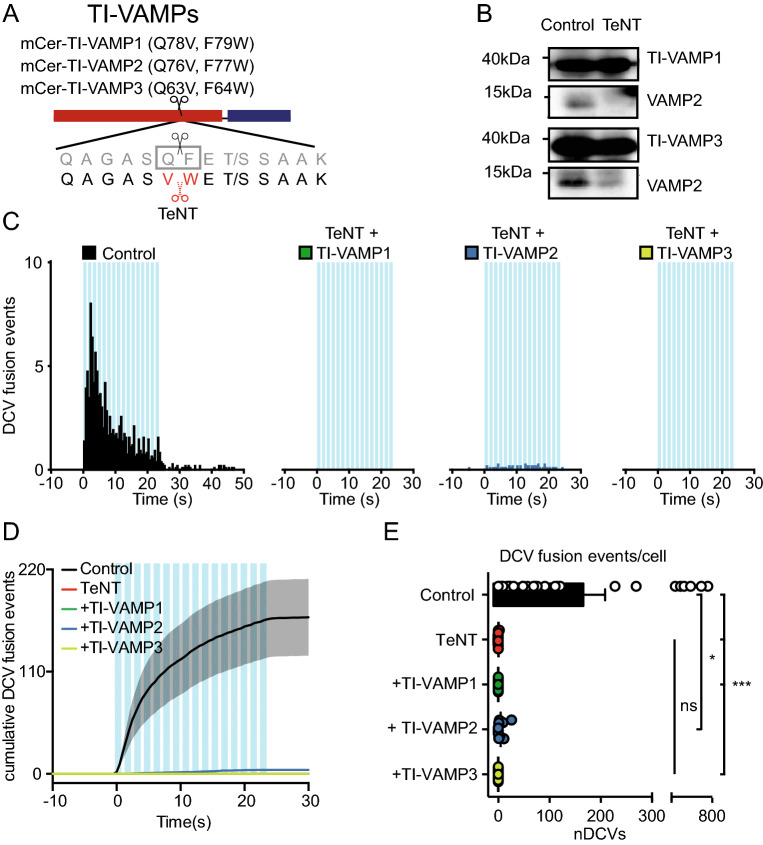
TI-VAMP1, TI-VAMP2 and TI-VAMP3 do not rescue DCV fusion in TeNT treated neurons. (**A**) Schematic representation of TI-VAMPs. Q78V, F79W (VAMP1), Q76V, F77W (VAMP2), Q63V, F64W (VAMP3) substitutions interfere with TeNT cleavage. N-terminal mCerulean (mCer) allows detection of the construct in live imaging experiments. (**B**) Western blot of neuronal lysate (DIV 16) infected with TI-VAMP1 or TI-VAMP3 (DIV 2), and control construct or TeNT (DIV 14), incubated with antibody against VAMP1, VAMP2 and VAMP3 (original blots are shown in Figure S4). (**C**) Histogram of DCV fusion events per cell in control, TeNT + TI-VAMP1, TeNT + TI-VAMP2, and TeNT + TI-VAMP3 infected neurons (infected with NPY-pHluorin at DIV 2, TI-VAMPs at DIV 0–4 and 8–9 and TeNT at DIV 8–9, imaged at DIV 14). Blue bars indicate 16 trains of 50 AP at 50 Hz interspaced by 0.5 s. (**D**) Cumulative plot of DCV fusion events of conditions as in C. Shaded area represents SEM. (**E**) Average DCV fusion events per cell for control (n = 27, N = 4), TeNT (n = 12, N = 4), TeNT + TI-VAMP1 (n = 11, N = 2), TeNT + TI-VAMP2 (n = 12, N = 2) and TeNT + TI-VAMP3 (n = 11, N = 2) infected neurons. Kruskal–Wallis with Dunn's correction: * p = 0.026, *** p < 0.0001, non-significant (ns) *p* > 0.05. Bars represent mean + SEM. Detailed statistics are shown in Supplementary Table S1.

To determine whether one of the TeNT sensitive VAMPs is sufficient for DCV fusion and to assess functional redundancy between VAMP isoforms, we studied DCV exocytosis in TeNT treated neurons expressing TeNT insensitive versions of VAMP1, 2 or 3. VAMP1 and VAMP3 contain the same cleavage sequence as VAMP2^19^, which were mutated in a similar fashion, obtaining TI-VAMP1 (VAMP1 Q78V, F79W) and TI-VAMP3 (VAMP3 Q63V, F64W; Fig. 3A). Hippocampal neurons were infected with TI-VAMP1, TI-VAMP2 or TI-VAMP3, together with NPY-pHluorin, and TeNT or a control construct. All three TI-VAMPs were resistant to TeNT cleavage, while endogenous VAMP2 was efficiently cut (Figs. 2B and 3B). Surprisingly, none of the three TI-VAMP proteins could restore DCV exocytosis in TeNT expressing neurons (DCV fusion events in control: 170.1 ± 41.3, TeNT: 0.17 ± 0.17, TeNT + TI-VAMP1: 0.0 ± 0.0, TeNT + TI-VAMP2: 4.6 ± 2.2, TeNT + TI-VAMP3: 0.0 ± 0.0; Fig. 3C–E). Only TI-VAMP2 infected neurons showed some DCV fusion, but not significantly more than TeNT treated neurons (TeNT vs TI-VAMP2: *p* = 0.38; Fig. 3C–E). We did not detect a single event in TeNT + TI-VAMP1 or TeNT + TI-VAMP3 infected neurons (Fig. 3C–E). In conclusion, and in contrast to SV vesicle fusion, neither TI-VAMP1, TI-VAMP2 nor TI-VAMP3 are sufficient to support DCV exocytosis in TeNT treated neurons.

### VAMP1 and VAMP2 both travel with DCVs

**Figure 4:**
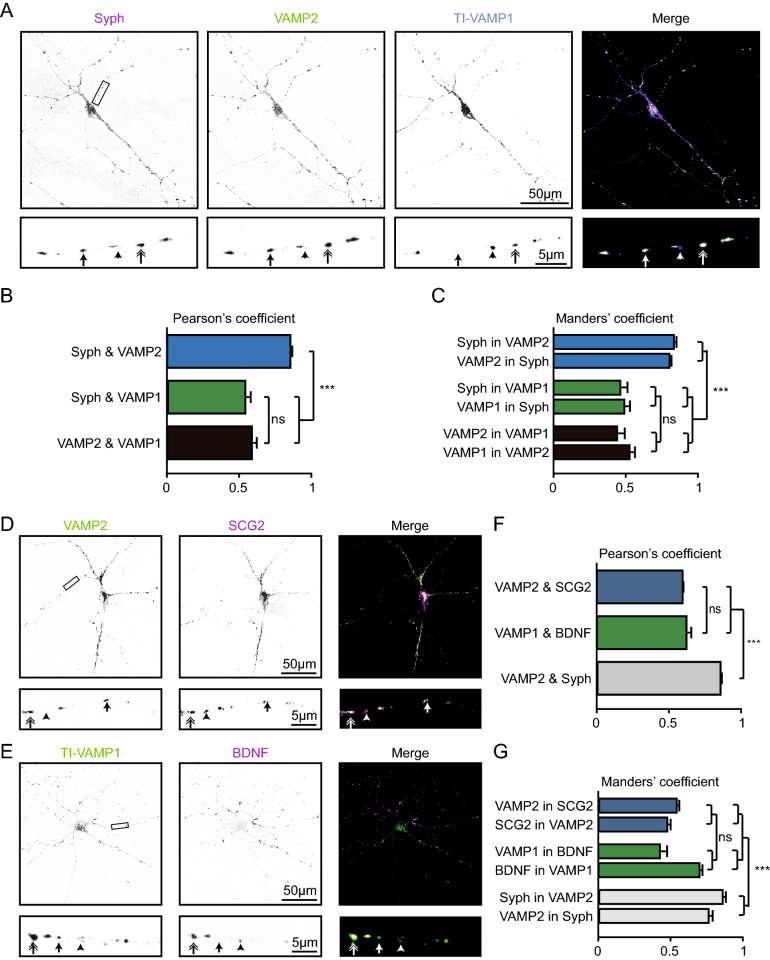
VAMP1 and VAMP2 co-localize with endogenous DCV markers. **(A)** Hippocampal neuron (DIV 11) infected with TI-VAMP1 (DIV 2) and stained for Synapthophysin (Syph, magenta), VAMP2 (green) or TI-VAMP1 (blue). Lower panels are a zoom of the upper panel. Arrowhead: VAMP1-positive punctum, arrow: VAMP2 and Syph co-localization, double arrow: VAMP1, VAMP2 and Syph co-localization. (**B**) Pearson's coefficient of Syph and VAMP1 or VAMP2, and of VAMP1 and VAMP2. One-way ANOVA with post hoc Tukey's test: Syph-VAMP2 vs syph-VAMP1/VAMP2-VAMP1: *p* < 0.0001 (***), rest is non-significant (ns). (**C**) Mander's coefficient of the co-localisation of Syph, VAMP1 and VAMP2. One-way ANOVA with post hoc Tukey's test: Syph in VAMP2/VAMP2 in Syph vs Syph in VAMP1/VAMP1 in Syph and vs VAMP2 in VAMP1/VAMP1 in VAMP2, *p* = 0.0001 (***), rest is non-significant (ns). (**D**) Hippocampal neuron (DIV 11) stained for VAMP2 (green) and secretogranin II (SCG2, magenta). Lower panels indicate the zoom of upper panels. Arrowhead: SCG2 punctum, arrow: VAMP2 punctum, double arrow: VAMP2 and SCG2 co-localization. (**E**) Hippocampal neuron (DIV 11) stained for VAMP1 (green) and BDNF (magenta). Lower panels indicate the zoom of upper panels. Arrowhead: BDNF punctum, arrow: VAMP1 punctum, double arrow: VAMP1 and BDNF co-localization. (**F**) Pearson's coefficient of VAMP2 and SCG2, VAMP1 and BDNF, and VAMP2 and Syph. One-way ANOVA with post hoc Tukey's test: VAMP2-SCG2/VAMP1-BDNF vs VAMP2-Syph: *p* < 0.0001 (***), VAMP2-SCG2 vs VAMP1-BDNF: *p* = 0.69 (ns). (**G**) Mander's coefficient of the co-localization of VAMP2 and SCG2, VAMP1 and BDNF, and VAMP2 and Syph. One-way ANOVA with post hoc Tukey’s test: VAMP2 in SCG2/SCG2 in VAMP2 vs Syph in VAMP2/VAMP2 in Syph and VAMP1 in BDNF/BDNF in VAMP1 vs Syph in VAMP2/VAMP2 in Syph: *p* < 0.0001 (***) rest is non-significant (ns). Detailed statistics are shown in Supplementary Table S1.

To examine which TeNT sensitive VAMP proteins localize to DCVs, we studied co-localization of VAMP1 and VAMP2 with canonical DCV and SV markers in hippocampal neurons. We did not include VAMP3 since the endogenous protein was not detected in neurons (Fig. 1C)^8,17^. VAMP1 expression in single hippocampal neurons was often below detection levels (data not shown) as reported before^9^. We therefore overexpressed TI-VAMP1. Punctate TI-VAMP1 localized with VAMP2 and the synaptic marker synaptophysin 1 (Syph, double arrow), without VAMP2 and Syph (arrowhead), or was absent from Syph and VAMP2 positive puncta (arrow, Fig. 4A). As expected, punctate VAMP2 staining strongly co-localized with Syph (Pearson's coefficient: 0.86 ± 0.02)^38^. In contrast, only a subset of TI-VAMP1 puncta overlapped with VAMP2 or Syph puncta (Pearson's coefficient: VAMP2–VAMP1: 0.59 ± 0.03, Syph-VAMP1: 0.55 ± 0.03, Fig. 4B, C).

TI-VAMP1 and endogenous VAMP2 puncta were found with (double arrow) or without (arrow) the DCV markers secretogranin II (SCG2, Fig. 4D) or brain derived neurotrophic factor (BDNF, Fig. 4E). In addition, both DCV markers were found without detectable TI-VAMP1 or VAMP2 (arrowhead, Fig. 4D, E). TI-VAMP1 and VAMP2 co-localized to a similar extent with respectively BDNF and SCG2 (Pearson's coefficient: VAMP2-SCG2: 0.60 ± 0.02, TI-VAMP1-BDNF: 0.62 ± 0.03, Fig. 4D–G). Co-localization of TI-VAMP1 and VAMP2 with DCV markers was lower compared to VAMP2 and Syph co-localization (Pearson's coefficient: VAMP2-Syph: 0.85 ± 0.02, Fig. 4F–G). In conclusion, VAMP2 is highly enriched at the synapse and both VAMP1 and VAMP2 partly co-localize with endogenous DCV markers.

**Figure 5:**
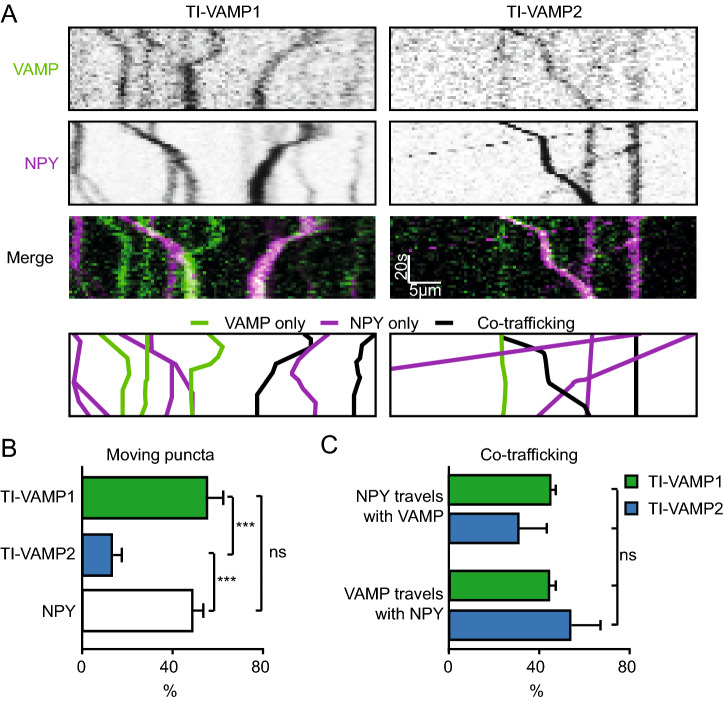
TI-VAMP1 and TI-VAMP2 co-travel with DCVs. (**A**) Representative kymographs of neurons co-infected (at DIV 4–6) with TI-VAMP1 and NPY-mCherry (left), or TI-VAMP2 and NPY-mCherry (right), imaged at DIV 11–13. VAMP kymographs in green, NPY kymographs in magenta. The bottom panels are graphic representations of the kymographs to show the quantification of the tracks: VAMP only (green), NPY only (magenta) or co-trafficking/localization (black). (**B**) Percentage moving (diagonal line in kymograph) TI-VAMP1 (n = 16, N = 2) TI-VAMP2 (n = 14, N = 2), and NPY (n = 30, N = 2) puncta. 1-way ANOVA with post-hoc Tukey’s test: VAMP2 vs VAMP1/NPY: *p* = 0.001 (***). (**C**) Percentage of moving NPY puncta with VAMP1 and VAMP2, and moving VAMP1 and VAMP2 puncta with NPY. 1-way ANOVA: *p* = 0.48 non-significant (ns). Bars represent mean + SEM. Detailed statistics are shown in Supplementary Table S1.

Since VAMP2 and to a lesser extent VAMP1 are present at synapses where both SVs and DCVs reside^2,26^ we were unable to assess co-localization with DCV markers at synapses. We therefore studied co-transport of DCVs and VAMP proteins in hippocampal neurons infected with the DCV marker NPY-mCherry and TI-VAMP1 or TI-VAMP2. A subset of NPY and TI-VAMP-positive puncta moved during time-lapse recordings of neurites (diagonal lines, Fig. 5A). More TI-VAMP2 puncta were stationary compared to TI-VAMP1 and NPY (moving puncta: TI-VAMP1: 56 ± 6.3%, TI-VAMP2: 13 ± 4.4%, NPY: 49 ± 4.8%; Fig. 5B). NPY puncta travelled with TI-VAMP1 or TI-VAMP2 (NPY with TI-VAMP1: 44 ± 3%, NPY with TI-VAMP2: 31 ± 13%) and, vice versa, mobile TI-VAMP puncta travelled together with DCVs (TI-VAMP2 with NPY: 54 ± 14%, TI-VAMP1 with NPY: 44 ± 3%), with no difference between the percentage of co-trafficking (Fig. 5C). In conclusion, TI-VAMP2-puncta are more often stationary than TI-VAMP1-puncta, and both TI-VAMP1 and TI-VAMP2 travel together with neuronal DCVs.

### Cleaved VAMP2 does not act as dominant negative for DCV fusion

**Figure 6:**
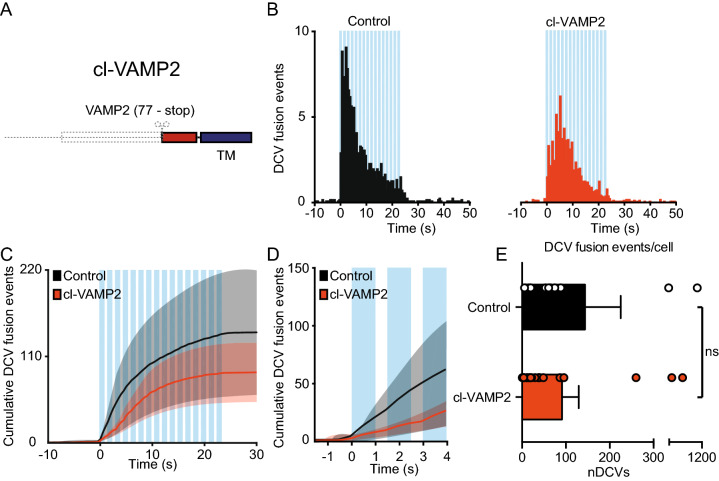
Cleaved VAMP2 does not act as a dominant negative for DCV fusion. **(A)** Schematic representation of the cleaved VAMP2 construct (cl-VAMP2) with the transmembrane domain remaining (blue). (**B**) Histogram of DCV fusion events in control (black) and cl-VAMP2 infected neurons (infected at DIV 8 with NPY-pHluorin and a control or cl-VAMP2 construct and imaged at DIV 14–16, orange). (**C**) Cumulative plot of DCV fusion events in control construct and cl-VAMP2 infected neurons. Shaded area represents SEM. Blue bars indicate 16 trains of 50 AP at 50 Hz interspaced by 0.5 s. (**D**) Cumulative DCV fusion events in control and cl-VAMP2 infected neurons during the first 3 bursts of stimulation. Blue bars indicate trains of 50 AP at 50 Hz interspaced by 0.5 s. (**E**) Average DCV fusion events per cell for control (n = 13, N = 3) and cl-VAMP2 (n = 20, N = 3) infected neurons. Mann–Whitney U test: p = 0.29 non-significant (ns). Bars represent mean + SEM. Detailed statistics are shown in Supplementary Table S1.

Since none of the TI-VAMP constructs restored DCV fusion, we investigated the possibility that the transmembrane domain of the endogenous VAMPs, which remains after TeNT cleavage, inhibits DCV fusion. Therefore, we designed cleaved (cl)-VAMP2 (77-stop, IRES-mCherry) corresponding to the transmembrane domain remaining after TeNT cleavage (Fig. 6A). Hippocampal neurons co-infected with NPY-pHluorin and cl-VAMP2 or a control construct showed a similar number of DCV fusion events after stimulation (control: 144 ± 82, cl-VAMP2: 91 ± 38; Fig. 6B–E). A cumulative plot of all fusion events (Fig. 6C) suggested a possible difference in the initial number of fusing DCVs (first 3 stimulation bursts). However, further inspection indicated no significant differences (DCV fusion events in the first 3 bursts control: 56.5 ± 41.7, cl-VAMP2: 23.6 ± 10.1; Fig. 6D). In conclusion, the TeNT-cleaved VAMP2 transmembrane fragment does not inhibit DCV fusion.

### Protease defective TeNT does not block DCV fusion

**Figure 7:**
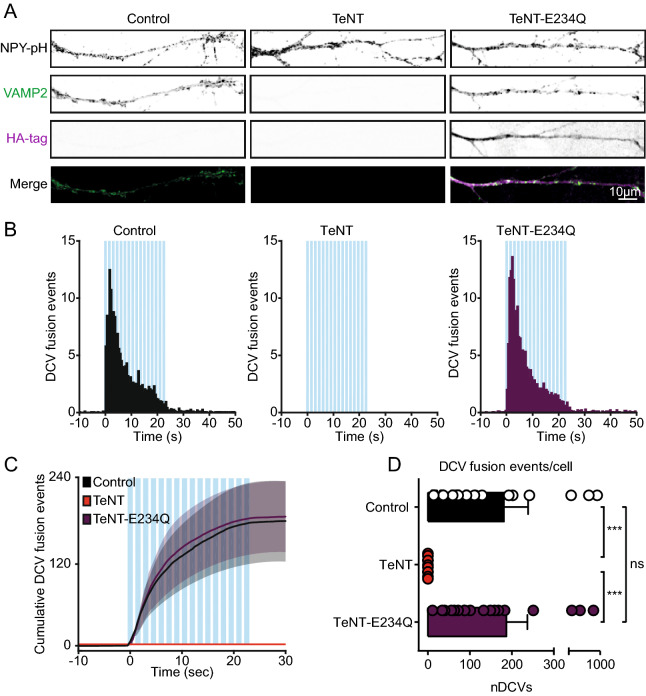
Protease defective TeNT does not block DCV fusion. (**A**) Representative images of DIV 14 neurites infected (DIV 10) with NPY-pHluorin and control, TeNT or TeNT-E234Q, stained for VAMP2 and the HA-tag. Only TeNT-E234Q was N-terminally tagged with HA. Merge indicates overlay of VAMP2 (green) and HA-tag (magenta) only. (**B**) Histograms of DCV fusion events in control (black), TeNT (red) or TeNT-E234Q (purple) infected hippocampal neurons (infected with NPY-pHluorin at DIV 9 and control, TeNT or TeNT-E234Q at DIV 10, imaged at DIV 14). Blue bars indicate 16 trains of 50 AP at 50 Hz interspaced by 0.5 s. (**C**) Cumulative plot of DCV fusion events of conditions as in B. Shaded area represents SEM. (**D**) Average DCV fusion events per cell for control (n = 19, N = 2), TeNT (n = 11, N = 2) and TeNT-E234Q (n = 18, N = 2) infected neurons. Kruskal–Wallis with Dunn's correction: ***p < 0.10* 10^−3^, non-significant (ns) *p* > 0.05. Bars represent mean + SEM. Detailed statistics are shown in Supplementary Table S1.

Although the TI-VAMP constructs are insensitive to TeNT cleavage (Figs. 2B, 3B), the residues required for VAMP recognition by TeNT^39^ were not affected^24^. As a consequence, TeNT may still bind to the TI-VAMP constructs and potentially block DCV fusion via steric hindrance. In addition, TeNT expression is known to activate transglutaminases which could indirectly affect DCV fusion^40,41^. To test the potential effects of steric hindrance or transglutaminase activation we designed a proteolytically inactive TeNT construct by substituting glutamic acid 234 for glutamine (TeNT-E234Q)^21,42^. The construct was N-terminally tagged with an HA-tag to validate expression and placed in an IRES-mCherry vector to visualize expression during live-cell experiments. Hippocampal neurons infected with NPY-pHluorin and a control, TeNT or TeNT-E234Q construct were stained for the HA-tag and VAMP2 (Fig. 7A). We found no loss of VAMP2 staining after lentiviral infection with TeNT-E234Q (Fig. 7A). These results confirm TeNT-E234Q to be proteolytically inactive^21,42^. Next, we studied DCV fusion in neurons co-infected NPY-pHluorin and a control, TeNT or the TeNT-E234Q construct. We found no difference in DCV fusion events between control or TeNT-E234Q infected neurons (DCV fusion events control: 181.1 ± 57.6, TeNT: 0 ± 0, TeNT-E234Q: 187.1 ± 50.1, Fig. 7B–D). To conclude, these results suggest that TeNT does not block DCV fusion via steric hindrance or transglutaminase activation.

### VAMP1 is not required for DCV fusion

**Figure 8:**
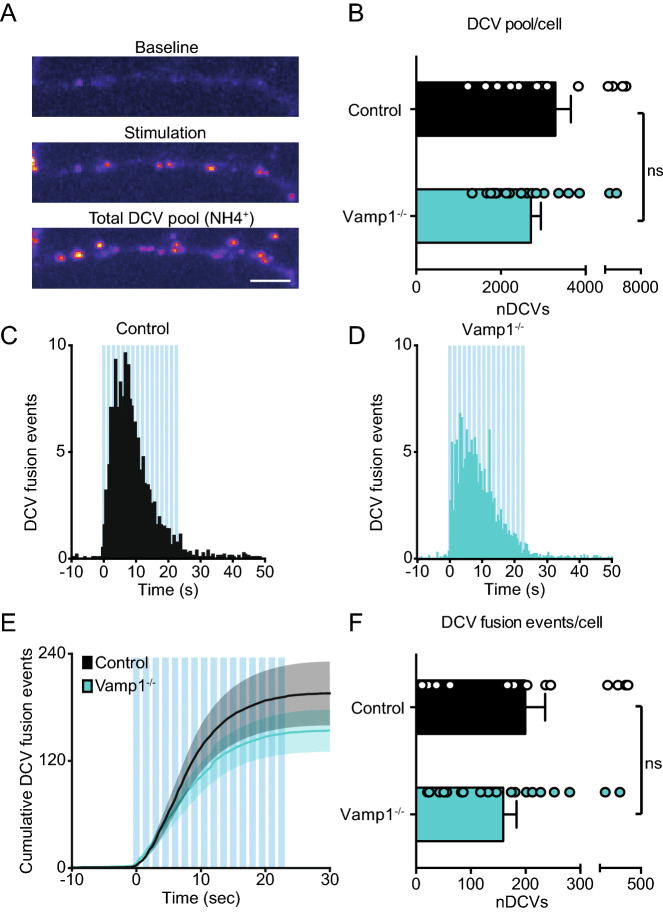
VAMP1 is not required for DCV fusion. (**A**) Panels show a neurite before stimulation (Baseline), during stimulation displaying fusion events (Stimulation), and the total number of NPY-pHluorin labeled DCVs upon NH4^+^ perfusion (Total DCV pool). Scale bar 5 µm. (**B**) Total number of NPY-pHluorin (infected at DIV 9) labeled DCVs (imaged at DIV 14) in control (n = 16, N = 3) and *vamp1*^*–/–*^ (n = 19, N = 3) neurons. Unpaired Student's t-test: *p* = 0.18 (ns). (**C**, **D**) Histograms of DCV fusion events in control (**C**) and *vamp1*^*–/–*^ (**D**) hippocampal neurons. (**E**) Cumulative DCV fusion events in control and *vamp1*^*–/–*^ hippocampal neurons. Shaded area represents SEM. Blue bars indicate 16 trains of 50 AP at 50 Hz interspaced by 0.5 s. (**F**) Average DCV fusion events per cell for control (n = 16, N = 3) and *vamp1*^*–/–*^ (n = 19, N = 3) neurons. Unpaired Student's *t*-test: *p* = 0.35 non-significant (ns). Bars represent mean + SEM. Detailed statistics are shown in Supplementary Table S1.

After budding from the trans Golgi network (TGN), DCVs undergo a process of maturation that is suggested to include homotypic fusion^43–47^ or fusion with sorting organelles^48^. Since not one single TI-VAMP (1, 2 or 3) construct could restore DCV fusion after TeNT treatment, we hypothesized that DCVs require VAMP1 and VAMP2 sequentially after budding of from the TGN. Therefore, we studied DCV exocytosis in the *vamp1lew (*lethal wasting, hereafter referred to as *vamp1*^*–/–*^) mouse that lacks VAMP1^25^. To exclude DCV biogenesis defects in *vamp1*^*–/–*^ hippocampal neurons, we analyzed the number of NPY-pHluorin puncta following NH_4_^+^ perfusion (Fig. 8A). No differences in the number of NPY puncta were found in *vamp1*^*–/–*^ neurons compared to wildtype littermate controls (DCV puncta control: 3,288 ± 364.3, *vamp1*^*–/–*^: 2,708 ± 237.2, Fig. 8B). Next, we compared DCV fusion of *vamp1*^*–/–*^ vs control neurons using NPY-pHluorin. We found no reduction of DCV fusion events in *vamp1*^*–/–*^ neurons compared to control neurons (DCV fusion events control: 199.4 ± 36.3, *vamp1*^*–/–*^: 158.9 ± 24.6, Fig. 8C–F). In conclusion, VAMP1 is not required for DCV fusion.

## Discussion

In this study, we assessed the role of VAMP proteins in neuronal DCV exocytosis in mouse hippocampal neurons. TeNT treatment, which cleaves VAMP1, 2 and 3, abolished SV and DCV fusion, as expected^10,19,20,23,36,37^. TI-VAMP2 restored SV fusion, but not DCV fusion in TeNT infected neurons. TI-VAMP1 and TI-VAMP3 also failed to restore DCV exocytosis in TeNT treated neurons. Hence, TeNT treatment combined with TI-VAMP2 expression differentially affects DCV and SV fusion.

We explored two possibilities for this unexpected difference between the two secretory pathways. First, we hypothesized that the cleaved transmembrane part of VAMP2, which could remain on the vesicle after TeNT cleavage, prevents targeting of enough TI-VAMP2 molecules to support DCV fusion despite sufficient targeting to SVs. However, overexpression of the cleaved transmembrane VAMP2 fragment did not block DCV fusion in WT neurons (Fig. 6). We therefore conclude that the lack of rescue of DCV fusion by TI-VAMP2 after TeNT cleavage cannot be explained by steric hindrance of this fragment. Secondly, we explored the possibility that DCVs require both VAMP1 and VAMP2 in a sequential scenario involving an upstream step before fusing with the plasma membrane. We considered VAMP1 to play a role in DCV fusion because VAMP1 deficiency reduces spontaneous and evoked synaptic transmission in the mouse neuromuscular junction^11^. In VAMP2 KO neurons, residual synaptic transmission correlates with VAMP1 expression levels, and silencing of VAMP1-expression further reduces synaptic transmission^9^. Hence, VAMP1 is expressed in neurons and functions in synaptic transmission in the peripheral and central nervous system. Furthermore, VAMP1 is localized on DCVs in the rat spinal cord^49^ and involved in release of CGRP in rat trigeminal ganglionic neurons^14,15^. In addition, we found that overexpressed VAMP1 and VAMP2 travel together with DCVs to a similar extent in hippocampal neurons (Fig. 5). However, DCV fusion in *vamp1*^*–/–*^ hippocampal neurons was unaffected (Fig. 8), indicating that VAMP1 is not essential for DCV exocytosis. VAMP3 is undetectable in neurons^8,17^ (Fig. 1) and was therefore not considered in a sequential scenario. In addition, acute knockdown of VAMP1 or VAMP3 did not reduce BDNF release in cortical neurons^10^. Hence, because (1) VAMP1 is dispensable for DCV fusion; (2) VAMP3 is not detected in hippocampal neurons; and (3) TeNT only cleaves VAMP1, 2 and 3, we conclude that VAMP2 is the only known vSNARE suitable to support DCV fusion in these neurons.

However, TI-VAMP2 could not rescue DCV fusion in TeNT treated neurons despite similar expression levels as endogenous VAMP2 and rescuing SV fusion (Fig. 2). This could be due to a number of reasons. First, TeNT cleaves VAMPs that are present on the vesicles. SVs contain on average 75 VAMP molecules^50^ and in liposomes and chromaffin cells, vesicles are estimated to require one to three SNARE complexes for fusion^51,52^. The number of VAMP molecules per DCV is unknown but DCV fusion pores in chromaffin cells are estimated to contain six to eight SNARE complexes^53,54^. Hence, DCVs likely require more VAMP molecules for fusion than SVs. Secondly, SVs locally recycle after fusion, increasing the likelihood of incorporating TI-VAMP2 into their membranes. DCVs do not recycle after fusion and the lifetime of a DCV after budding from the TGN is unknown. Therefore, incorporation of TI-VAMP2 molecules is likely slower. Although TI-VAMP1 and TI-VAMP2 travelled together with DCVs (Fig. 5), the number of TI-VAMP2 molecules per vesicle may be insufficient to support DCV fusion but sufficient for SV fusion after TeNT treatment. Taken together, loading of relatively less TI-VAMP2 on DCV and a higher demand for TI-VAMP2 molecules to support DCV fusion could contribute to the inability of TI-VAMP2 to rescue DCV fusion after TeNT treatment.

TeNT is also known to activate transglutaminases which is shown to have minor effects on synaptic transmission by itself^40,41^. However, transglutaminase activity could have a stronger negative effect on DCV fusion. Alternatively, the fact that TI-VAMP2 restores SV fusion, but not DCV fusion after TeNT treatment, might also be explained by steric hindrance of DCVs selectively. The residues required for VAMP2 recognition by TeNT^39^ were not affected by the mutations in the TI-VAMP2 construct. Therefore, TeNT still recognizes TI-VAMP2 and could affect the efficiency of fusion via steric hindrance upon binding TI-VAMP2. The effective window of TeNT proteolysis is before docking, before a SNARE complex is formed (reviewed by^19^). Since DCVs are typically not predocked at the plasma membrane^26^, TeNT might constantly bind VAMP molecules and prevent fusion via steric hindrance. In line with the potential effects of steric hindrance, our SV fusion experiments show a slower, though not significant, rise in sypHy signal during extensive stimulation in TeNT treated neurons (Fig. 2), possibly reflecting a negative role of TeNT binding to TI-VAMP2 on newly recruited vesicles. Alternatively, these results could be explained by transglutaminase activation^40,41^. However, treating neurons with TeNT-E234Q, which is still able to bind VAMPs and activate transglutaminases^21,40–42,55^, did not change the number of DCV fusion events (Fig. 7). These results exclude that transglutaminase activation or steric hindrance by TeNT binding alone can account for the lack of DCV fusion rescue by TeNT insensitive VAMP2.

In conclusion, the failure of TI-VAMP2 to restore DCV fusion after TeNT treatment indicates a novel mechanistic difference between the SV and DCV secretory pathways and can be used as a tool to selectively target DCV fusion leaving SV fusion unaffected.

## Materials and methods

### Biosafety

All experiments procedures were performed according to the local guidelines of the VU University/ VU University Medical Centre. For lentiviral work we followed all safety measures according to European legislation (ML-II, permit number: IG16-223-IIk).

### Plasmids

mCerulean-VAMP2 Q76V, F77W (TI-VAMP2)^24^ was purchased from Addgene. mCerulean-VAMP1 Q78V, F79W (TI-VAMP1) and mCerulean-VAMP3 Q63V, F64W (TI-VAMP3) were designed according to the TI-VAMP2 construct. Cleaved VAMP2 was cloned from TI-VAMP2 and cloned into an IRES-mCherry vector^56^. As a control we used scrambled RNA cloned into an IRES-mCherry vector. All constructs were cloned into pLenti vectors containing the neuron specific synapsin promoter. NPY-mCherry, NPY-pHluorin, synaptophysin-pHluorin (sypHy), and TeNT-IRES- mCherry were described before^35,56,57^. Proteolytically inactive TeNT^21,42^ was generated by substituting glutamic acid 234 for glutamine, N-terminally tagged with a HA-tag and cloned into an IRES-mCherry vector. lentiviral particles were produced as described before^58^.

### Laboratory animals, primary neuron cultures and infection

All animal experiments were approved by the animal ethical committee of the VU University/ VU University Medical Centre (license number: FGA 11-03 and AVD112002017824). Animals were housed and bred according to institutional and Dutch governmental guidelines and regulations. Wildtype mouse neurons were obtained from embryonic day 18 (E18) embryos, acquired by caesarean section of pregnant mice. Neurons from *Vamp1*^*lew/lew*^ ^25^ and wildtype control littermates were taken at P1. Primary neuron cultures were prepared as described before^29,59^. In short, dissected hippocampi and cortices were digested with 0.25% trypsin (Life Technologies) in Hanks’ balanced salt solution (Sigma) with 10 mM HEPES (Life Technologies) for 20 min at 37 °C. Hippocampi were washed, triturated and 1,000–2,000 neurons/well were plated on pre-grown micro-islands generated by plating 6,000 rat glia on 18 mm glass coverslips coated with agarose and stamped with a solution of 0.1 mg/ml poly-d-lysine (Sigma) and 0.7 mg/ml rat tail collagen (BD Biosciences)^60,61^. For western blots, cortices were washed, triturated and 300,000 neurons/well were plated on 6 well plates coated with a solution of 0.5*10^−3^% poly-l-ornithine and 2.5 μg/ml laminin (Sigma). For trafficking experiments, 1,000 neurons/well were plated on 18 mm glass coverslips coated with a solution of 0.5*10^−3^% poly-l-ornithine and 2.5 μg/ml laminin (Sigma). Neurons were kept in neurobasal medium supplemented with 2% B-27, 18 mM HEPES, 0.25% glutamax and 0.1% Pen-Strep (Life Technologies) at 37 °C and 5% CO_2_. Whole brain lysate was derived from brains from wildtype E18 embryos.

### Imaging

Neurons were imaged in Tyrode’s solution (2 mM CaCl_2_, 2.5 mM KCl, 119 mM NaCl, 2 mM MgCl_2_, 30 mM glucose, 25 mM HEPES; pH 7.4). Imaging was performed with a custom build microscope containing an imaging microscope (AxioObserver.Z1, Zeiss), 561 nm and 488 nm lasers, polychrome V, appropriate filter sets, 40 × oil objective (NA 1.3) and an EM-CCD camera (C9100-02; Hamamatsu, pixel size 200 nm). Images were acquired with AxioVision software (version 4.8, Zeiss). Electrical stimulation was performed with two parallel platinum electrodes placed around the neuron. 16 trains of 50 action potentials at 50 Hz, interspaced by 0.5 s, were initiated by a Master-8 (AMPI) and a stimulus generator (A-385, World Precision Instruments) delivered the 1 ms pulses of 30 mA. Tyrode’s with 50 mM NH_4_Cl (replacing 50 mM NaCl) was delivered by gravity flow through a capillary placed above the neuron. Experiments were performed at room temperature (21–25 °C).

For all DCV and SV fusion experiments (with NPY-pHluorin or sypHy, respectively), 200 images were acquired at 2 Hz. Neurons were selected if all indicated constructs were expressed (based on expression tags). After the first 30 s of baseline recording (the last 10 s are shown in the figures) neurons were stimulated with 16 burst of 50 AP at 50 Hz, interspaced by 0.5 s. After 90 s, neurons were superfused with NH_4_^+^ (not shown in the figures). Image acquisition, stimulation and NH_4_^+^ perfusion timing were set automatically via AxioVision software (version 4.8, Zeiss) and a Master-8 controlling the stimulus generator. For DCV co-trafficking experiments, 45 images were acquired at 0.5 Hz and 2 × 2 binning. For every image, two consecutive frames in both colour channels were acquired.

### Immunocytochemistry

Neurons were fixed in 2% formaldehyde (Merck) in phosphate-buffered saline (PBS; 137 mM NaCl, 2.7 mM KCl, 10 mM Na_2_HPO_4_, 1.8 mM KH_2_PO_4_, pH 7.4) for 10 min followed by 4% formaldehyde in PBS for 30 min. Cells were permeabilized in 0.5% TritonX-100 (Fisher Chemical) for 5 min and blocked with 0.1% TritonX-100 and 2% normal goat serum for 30 min. Incubation with primary antibodies were performed in 2 h at room temperature or overnight at 4 °C. Primary antibodies used were: polyclonal MAP2 (Abcam 1:1,000), monoclonal VAMP2 (Sysy, 1:2000), polyclonal GFP (bioconnect, 1:1,000), polyclonal SCG2 (Biodesign International, 1:500), monoclonal BDNF (DSHB, 1:4), polyclonal synaptophysin 1 (SySy, 1:1,000) polyclonal mCherry (GeneTex, 1:2000), polyclonal HA-tag (Abcam, 1:500). Alexa Fluor conjugated secondary antibodies (1:1,000; Invitrogen) were incubated for 1 h at room temperature. Coverslips were mounted in Mowiol and imaged on a Zeiss LSM 510 confocal laser-scanning microscope (40 × objective; NA 1.3) and LSM510 software (version 3.2 Zeiss) (Figs. 1, 4 and S1) or on a A1R Nikon confocal microscope with LU4A laser unit (40 × objective; NA 1.3) and NIS elements software (version 4.60, Nikon) (Fig. 7).

### Western blotting

Cortical neurons or whole brain lysate were run on an SDS-PAGE gel and transferred to a nitrocellulose membrane (Bio-rad). Membranes were blocked 2.5% BSA (Acros Organics) in PBS with 0.1% Tween-20, and subsequently incubated with polyclonal VAMP1 (Sysy; 1:1,000), monoclonal VAMP2 (Sysy; 1:2000) or polyclonal VAMP3 (Abcam, 1:1,000) antibodies overnight (4 °C) and 2 h with monoclonal actin (Chemicon; 1:10.000). Membranes were incubated with fluorescent IRDye secondary antibodies (1:5,000–1:15,000, LI-COR) and scanned with an Odyssey Fc imaging system (Li-COR Bioscience). Brightness and contrast was adjusted with ImageJ.

### Data analysis

Neurite length and branching, Sholl analysis, and number, size and intensity of NPY positive puncta were analyzed with SynD^28,62^ software (version 491) running in MATLAB (MathWorks, Inc.). In Fig. 6, time-lapse recordings during NH_4_^+^ perfusion, which un-quenched the NPY-pHluorin, were used to quantify the number of DCVs. Pearson's and Mander's coefficients for co-localization analysis were obtained using the JaCoP plugin^63^.

DCV exocytosis events were detected manually as sudden appearance of NPY-pHluorin positive puncta using ImageJ. For experiments with infection of multiple constructs only neurons that expressed all indicated constructs (based on expression tags) were used for analysis. NPY-pHluorin events were considered a fusion event if the maximal fluorescence was at least twice the SD above noise. Custom written MATLAB scripts were used to calculate the number and timing of fusion events. Quantification of moving puncta and co-trafficking was done manually. Kymographs were generated with ImageJ of a neurite stretch positive for both NPY-mCherry and VAMP. Kymograph lines were analyzed for stationary (vertical lines) and moving puncta (diagonal lines). Co-trafficking was determined by overlapping lines of moving puncta. SV exocytosis was measured with ImageJ in manually placed regions where NH_4_^+^ increased fluorescence, where the Fstim_max_ was the maximal response during stimulation relative to the maximal response during NH_4_^+^ superfusion.

### Statistics

Normal distributions for all datasets were assessed first using Shapiro–Wilk normality tests. To test more than 2 groups, we used one-way analysis of variance (ANOVA) followed by a post-hoc Tukey test to compare conditions when data was normally distributed or the Kruskal–Wallis test for non-parametric data followed by Dunn’s multiple comparisons test to compare conditions. To compare two groups, we used an unpaired Student's t-test in the case of normal distributed data (only for the total number of NPY-pHluorin labeled DCVs and the number of DCV fusion events between control and *vamp1*^*–/–*^ neurons, Fig. 6) or Mann–Whitney *U* tests for non-parametric data (all other cases). Data is represented as average with standard error of the mean (SEM). Dots in bar graphs indicate individual data points of single neurons. All data and statistical tests used are summarized in detail in Supplementary Table S1.

## Supplementary information

Supplementary file1 (PDF 1440 kb)

Supplementary file2 (BIB 125 kb)
