# Supplementary material for: Tetanus insensitive VAMP2 differentially restores synaptic and dense core vesicle fusion in tetanus neurotoxin treated neurons
Source: Sci Rep. 2020 Jul 2;10:10913. doi: 10.1038/s41598-020-67988-2 (PMC7331729; doi:10.1038/s41598-020-67988-2)
Supplement: Supplementary file 1 — Supplementary file1 (PDF 1440 kb) [file 41598_2020_67988_MOESM1_ESM.pdf]

Supplementary files

Tetanus neurotoxin differentially affects synaptic and dense core vesicle fusion in mouse hippocampal neurons

Rein I. Hoogstraaten[1#], Linda van Keimpema[1,3#], Ruud F. Toonen[1,*], Matthijs Verhage[1,2,*]

[1]*Department of Functional Genomics and* [2]*Clinical Genetics, Center for Neurogenomics and Cognitive Research, Vrije Universitieit (VU) Amsterdam and University Medical Center Amsterdam, de Boelelaan 1087, 1018 HV Amsterdam, The Netherlands*

[3]*Sylics (Synaptologics BV), PO box 71033, 1008 BA, Amsterdam, The Netherlands*

[#]These authors contributed equally

*To whom correspondence should be addressed: Matthijs Verhage, PhD (matthijs@cncr.vu.nl) or Ruud Toonen, PhD (ruud.toonen@cncr.vu.nl)

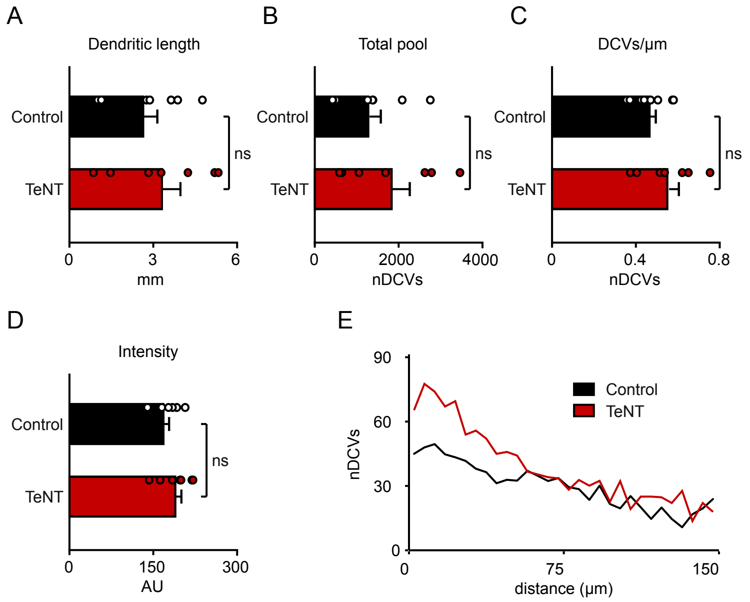

Supplemental figure 1: **Tetanus infection does not affect number, loading and localization of DCVs.** (**A**) Average dendritic length at DIV4 of control construct infected (n = 8, N = 2) or TeNT infected neurons (n = 7, N = 2, infected at DIV 0) stained for MAP2 and NPY-pHluorin (infected at DIV0). Unpaired Student's t-test: p = 0.42 (ns). (**B**) Average total DCV pool of control construct or TeNT

infected neurons. Unpaired Student's t-test: p = 0.28 (ns). (**C**) Average number of DCVs per dendritic length of control and TeNT infected neurons. Unpaired Student's t-test: p = 0.16 (ns). (**D**) Average intensity of DCVs of control and TeNT infected neurons. Unpaired Student's t-test: p = 0.16 (ns). (**E**) Scholl analysis of DCVs along the dendrites in control and TeNT infected neurons. Bars represent mean + SEM, individual neurons are represented by dots. Detailed statistics are shown in table S1.

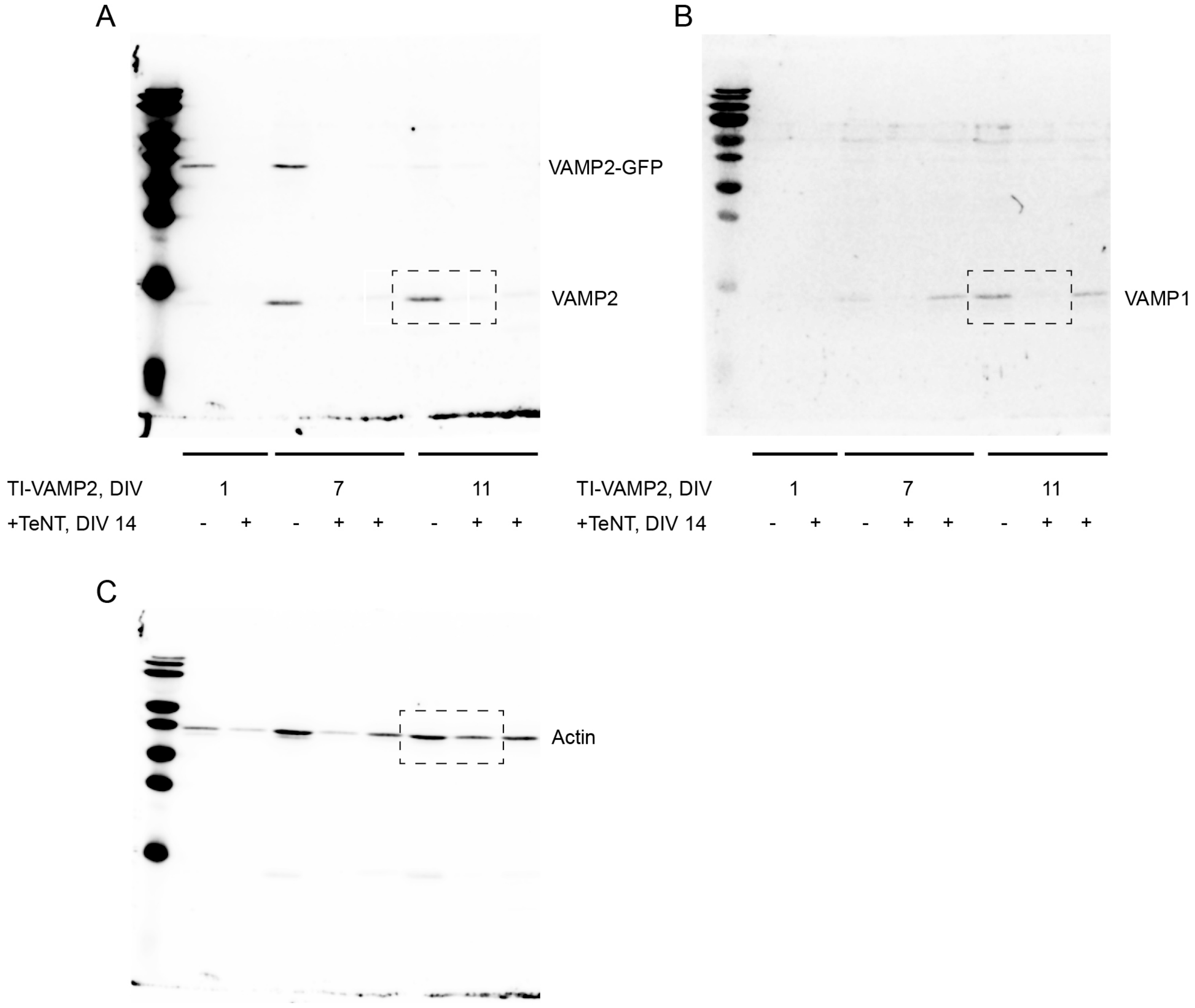

Supplemental figure 2: Original western blots Figure 1B. (**A**) Western blots of neuronal lysate (DIV 16) co-infected with TI-VAMP2-GFP (never used elsewhere, at the DIVs indicated) and with a control construct (-) or with TeNT (+) at DIV14. (**B**) Same blot as in A, stained for VAMP1. (**C**) Same blot as in A, stained for actin (in the same wavelength as A). Boxed area indicates the cropped area used in figure 1B. First lane indicates the marker in all with from top to bottom 180, 130, 70, 55, 40, 35, 25, 15 and 10 kDa.

A

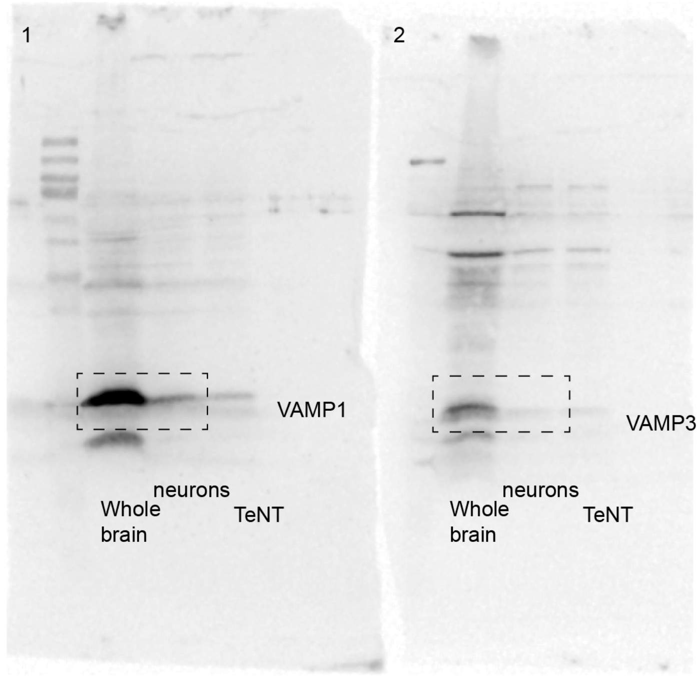

B

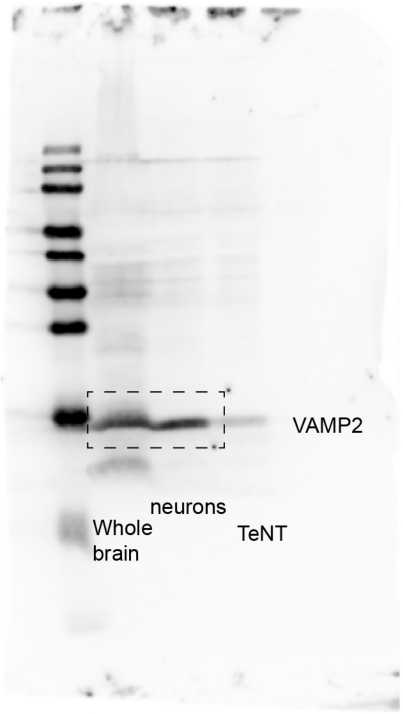

Supplemental figure 3: Original western blots Figure 1C. (**A**) Western blots of whole brain lysate or cultured neuronal lysate (DIV13) infected with TeNT (DIV 10) as indicated underneath each lane stained for VAMP1 (blot 1) or VAMP3 (blot 2). (**B**) Same blots as in A(1) showing VAMP2 staining. First lane indicates the marker in all with from top to bottom 180, 130, 70, 55, 40, 35, 25, 15 and 10 kDa.

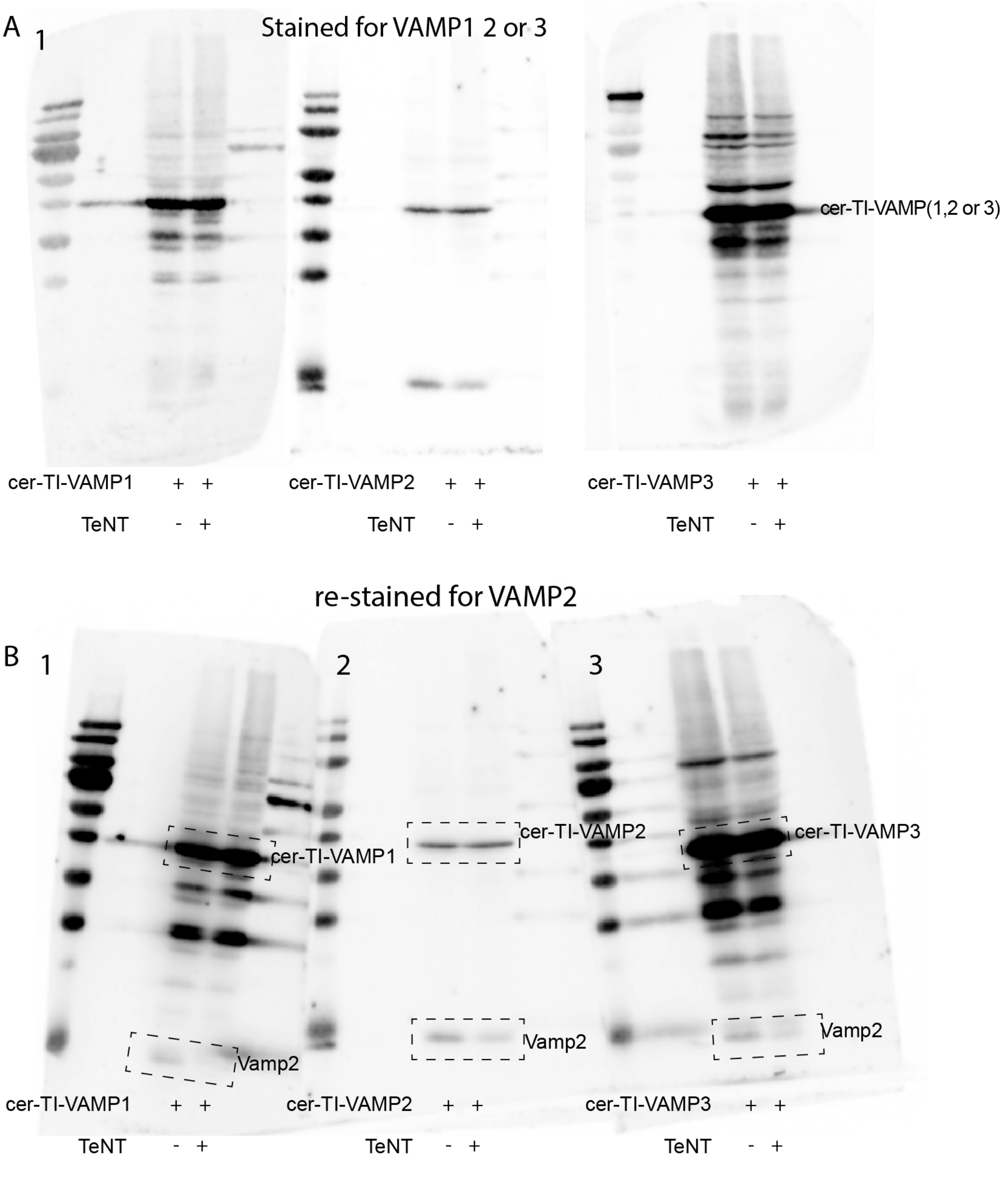

Supplemental Figure 4: Original western blots figure 2 and 3. (**A**) Western blots of neuronal lysate (DIV 16) infected with TI-VAMP1 (DIV 2) and stained for VAMP1 (blot 1), infected with TI-VAMP2 (DIV 2) and stained for VAMP2 (blot 2) or infected with TI-VAMP3 (DIV 2) and stained for VAMP3 (blot 3). All are co-infected (DIV 14) with a control construct (-) or TeNT (+) as indicated below. (**B**) Same blots as in A (re-)stained for VAMP2. Boxed areas in B indicates the cropped TI-VAMP and VAMP2 staining examples used in Fig. 2 and 3. The brightness and contrast is set to show both overexpressed TI-VAMP and endogenous VAMP2 signal. First lane indicates the marker in all with from top to bottom 180, 130, 70, 55, 40, 35, 25, 15 and 10 kDa.

Table S1: Data and statistics. Overview of all data and statistics for each figure. Dataset, condition, average and SEM, the number of independent weeks (N) and number of independent cells (n), the p values and statistical tests used are indicated. Statistical tests were two-tailed and used α = 0.05. * p < 0.05, ** p < 0.01, *** p < 0.001. For one-way ANOVA or Kruskal-Wallis tests the p-values are also indicated.

Dataset	condition	value (mean ± SEM)	N(n)	p value	Statistical test
DCV fusion Figure 1	Control	57 ± 19	3(21)	*** p = $4.5*10^{-7}$	Mann-Whitney U test
	TeNT	0.14 ± 0.08	3(21)		
SV fusion Figure 2	(1) Control	0.46 ± 0.05	2(6)	** p = 0.0067: 1 versus 2<br>* p = 0.0116: 2 versus 3<br>ns p > 0.99: 1 versus 3	Kruskal-Wallis (p = 0.0053) with Dunn's correction
	(2) TeNT	0.005 ± 0.0003	2(4)		
	(3) TeNT + TI-VAMP2	0.36 ± 0.05	2(17)		
DCV fusion Figure 3	(1) Control	170.1 ± 41.34	4(27)	*** p < 0.0001: 1 versus 2, 1 versus 3, 1 versus 5<br>* p = 0.026: 1 versus 4<br>ns, p >0.99: 2 versus 3, 2 versus 5, 4 versus 5, p = 0.38: 2 versus 4, p = 0.23: 3 versus 4, 3 versus 5	Kruskal-Wallis (p < 0. $1*10^{-3}$) with Dunn's correction
	(2) TeNT	0.17 ± 0.17	2(12)		
	(3) TeNT + TI-VAMP1	0 ± 0	2(11)		
	(4) TeNT + TI-VAMP2	4.58 ± 2.21	2(12)		
	(5) TeNT + TI-VAMP3	0 ± 0	2(11)		
Pearson's coefficient Figure 4B	(1) Syph-VAMP2	0.86 ± 0.02	1(10)	*** p < 0.0001: 1 versus 2, 1 versus 3<br>ns p = 0.59: 2 versus 3	One-way ANOVA (p = $0.3*10^{-9}$) with Tukey's correction
	(2) Syph-VAMP1	0.55 ± 0.03	1(10)		
	(3) VAMP2-VAMP1	0.59 ± 0.03	1(10)		
Mander's coefficient Figure 4C	(1) Syph in VAMP2	0.83 ± 0.02	1(10)	*** p < 0.0001: 1 and 2 vs 3 and 4, 1 and 2 versus 5 and 6<br>ns p = 0.99: 3 and 4 versus 5 and 6	One-way ANOVA (p = $0.14*10^{-12}$) with Tukey's correction
	(2) VAMP2 in Syph	0.80 ± 0.01	1(10)		
	(3) Syph in VAMP1	0.46 ± 0.05	1(10)		
	(4) VAMP1 in Syph	0.49 ± 0.04	1(10)		
	(5) VAMP2 in VAMP1	0.44 ± 0.05	1(10)		
	(6) VAMP1 in VAMP2	0.52 ± 0.04	1(10)		
Pearson's coefficient Figure 4F	(1) VAMP2-SCG2	0.60 ± 0.02	1(8)	ns p = 0.69: 1 versus 2,<br>*** p < 0.0001: 1 versus 3, 2 versus 3	One-way ANOVA (p = $0.14*10^{-8}$) with Tukey's correction
	(2) VAMP1-BDNF	0.62 ± 0.03	1(7)		
	(3) VAMP2-Syph	0.85 ± 0.02	1(8)		
Mander's coefficient Figure 4G	(1) VAMP2 in SCG2	0.52 ± 0.04	1(8)	ns p = 0.47: 1 and 2 versus 3 and 4<br>*** p < 0.0001: 1 and 2 versus 5 and 6, 3 and 4 versus 5 and 6	One-way ANOVA (p = $0.21*10^{-11}$) with Tukey's correction
	(2) SCG2 in VAMP2	0.48 ± 0.05	1(8)		
	(3) VAMP1 in BDNF	0.43 ± 0.05	1(7)		
	(4) BDNF in VAMP1	0.70 ± 0.04	1(7)		

	(5) Syph in VAMP2	0.84 ± 0.02	1(8)		
	(6) VAMP2 in Syph	0.77 ± 0.03	1(8)		
% moving puncta Figure 5	(1) % moving TI-VAMP1	56 ± 6.3 %	2(16)	*** p = 0.001: 1 versus 2, 3 versus 2<br>ns p = 0.58: 1 versus 3	One-way ANOVA (p = $0.13*10^{-6}$) with Tukey's correction
	(2) % moving TI-VAMP2	13 ± 4.4 %	2(14)		
	(3) % moving NPY	49 ± 4.8 %	2(30)		
Co-travel Figure 5	NPY with TI-VAMP1	44 ± 3 %	2	ns p = 0.48	One-way ANOVA (p = 0.48)
	NPY with TI-VAMP2	31 ± 13 %	2		
	TI-VAMP1 with NPY	44 ± 3 %	2		
	TI-VAMP2 with NPY	54 ± 14 %	2		
DCV fusion Figure 6	Control	143.6 ± 81.45	3(13)	ns p = 0.29	Mann-Whitney U test
	cl-VAMP2	91.3 ± 37.93	3(20)		
DCV fusion first 3 bursts Figure 6	Control	56.54 ± 41.69	3(13)	ns p = 0.57	Mann-Whitney U test
	cl-VAMP2	22.40 ± 10.06	3(20)		
number of DCVs Figure 7	Control	1219 ± 364.3	3(16)	ns p = 0.18	Unpaired Student's t-test
	vamp1-/-	1321 ± 237.2	3(19)		
DCV fusion events Figure 7	Control	199.4 ± 36.33	3(16)	ns p = 0.35	Unpaired Student's t-test
	vamp1-/-	158.9 ± 24.59	3(19)		
DCV fusion events Figure 8	(1) Control	181.1 ± 57.60	2(19)	*** p < 0.0001: 1 versus 2, 2 versus 3<br>ns p > 0.99	Kruskal-Wallis (p < 0. $1*10^{-3}$) with Dunn's correction
	(2) TeNT	0 ± 0	2(11)		
	(3) TeNT-E234Q	187.1 ± 50.1	2(18)		
Dendritic length Figure S1	Control	2.6 ± 0.5 mm	2(8)	ns p = 0.42	Unpaired Student's t-test
	TeNT	3.3 ± 0.5 mm	2(7)		
Total pool Figure S1	Control	1279 ± 296	2(8)	ns p = 0.28	Unpaired Student's t-test
	TeNT	1836 ± 430	2(7)		
DCVs/μm Figure S1	Control	0.46 ± 0.03	2(8)	ns p = 0.16	Unpaired Student's t-test
	TeNT	0.55 ± 0.05	2(7)		
Intensity Figure S1	Control	170 ± 8.9	2(8)	ns p = 0.17	Unpaired Student's t-test
	TeNT	190 ± 11	2(7)		
